# Image Quality in Adaptive Optics Optical Coherence Tomography of Diabetic Patients

**DOI:** 10.3390/diagnostics15040429

**Published:** 2025-02-10

**Authors:** Elisabeth Brunner, Laura Kunze, Wolfgang Drexler, Andreas Pollreisz, Michael Pircher

**Affiliations:** 1 Center for Medical Physics and Biomedical Engineering, Medical University of Vienna, Waehringer Guertel 18-20, A-1090 Wien, Austria; a.elisabeth.brunner@meduniwien.ac.at (E.B.); wolfgang.drexler@meduniwien.ac.at (W.D.); 2 Department of Ophthalmology and Optometry, Medical University of Vienna, Waehringer Guertel 18-20, A-1090 Wien, Austria; laura.kunze@meduniwien.ac.at (L.K.); andreas.pollreisz@meduniwien.ac.at (A.P.)

**Keywords:** adaptive optics, optical coherence tomography, image quality, diabetic retinopathy

## Abstract

**Background/Objectives**: An assessment of the retinal image quality in adaptive optics optical coherence tomography (AO-OCT) is challenging. Many factors influence AO-OCT imaging performance, leading to greatly varying imaging results, even in the same subject. The aim of this study is to introduce quantitative means for an assessment of AO-OCT image quality and to compare these with parameters retrieved from the pyramid wavefront sensor of the system. **Methods**: We used a spectral domain AO-OCT instrument to repetitively image six patients suffering from diabetic retinopathy over a time span of one year. The data evaluation consists of two volume acquisitions with a focus on the photoreceptor layer, each at five different retinal locations per visit; 7–8 visits per patient are included in this data analysis, resulting in a total of ~420 volumes. **Results**: A large variability in AO-OCT image quality is observed between subjects and between visits of the same subject. On average, the image quality does not depend on the measurement location. The data show a moderate correlation between the axial position of the volume recording and image quality. The correlation between pupil size and AO-OCT image quality is not linear. A weak correlation is found between the signal-to-noise ratio of the wavefront sensor image and the image quality. **Conclusions**: The introduced AO-OCT image quality metric gives useful insights into the performance of such a system. A longitudinal assessment of this metric, together with wavefront sensor data, is essential to identify factors influencing image quality and, in the next step, to optimize the performance of AO-OCT systems.

## 1. Introduction

Adaptive optics optical coherence tomography (AO-OCT) is a powerful imaging modality that allows for cellular imaging in the living human retina [1,2,3]. Using this technology enables the visualization of various cell types, such as cone photoreceptors [4,5], rod photoreceptors [6,7], retinal pigment epithelium cells [8,9,10], ganglion cells [11,12] and microglia [13,14]. Despite the success of this imaging modality in healthy subjects, the translation to clinically relevant cases is rather challenging. Earlier reports showed imaging results in age-related macular degeneration [15], glaucoma [16,17,18] and diabetic retinopathy [19,20]. However, the achieved image quality seems significantly lower than in healthy subjects and shows a large variability (possibly due to cataract, smaller pupil size or more pronounced motion).

A similar variability in image quality was found in adaptive optics scanning laser ophthalmoscopy (AO-SLO) [21]. For this technique, various imaging metrics were introduced to quantitatively assess, for example, the visibility of cone photoreceptors. Another related study investigated the variability of AO-SLO image quality over time in subjects without pupil dilation [22]. While these papers emphasize the need for a quantitative assessment of imaging quality in AO-assisted imaging, similar studies for AO-OCT are still missing.

In this work, we investigate the varying AO-OCT image quality in diabetic patients at different imaging locations of the retina and over several visits. We further analyze our corresponding pyramid wavefront sensor data to report on AO correction quality, pupil size, central pupil position and signal-to-noise ratio (SNR) of the pupil images. Finally, we provide a correlation analysis between these parameters and the AO-OCT image quality.

## 2. Materials and Methods

The AO-OCT system is described in detail elsewhere [14]. In brief, the instrument records 250,000 A-scans per second and covers an imaging area of ~4° × 4° field of view on the retina. The imaging wavelength of 840 nm with a bandwidth of 50 nm provides 4.5 µm axial resolution in tissue. AO correction is based on a pyramid wavefront sensor that is illuminated by part of the imaging light returning from the retina [12]. The AO-loop is operated at 25 Hz. As a correction device, a deformable mirror (DM) consisting of 69 elements is used. The estimated diffraction-limited transverse resolution of the system is 2 µm. The recording time of a single volume is ~2.5 s.

**Figure 1:**
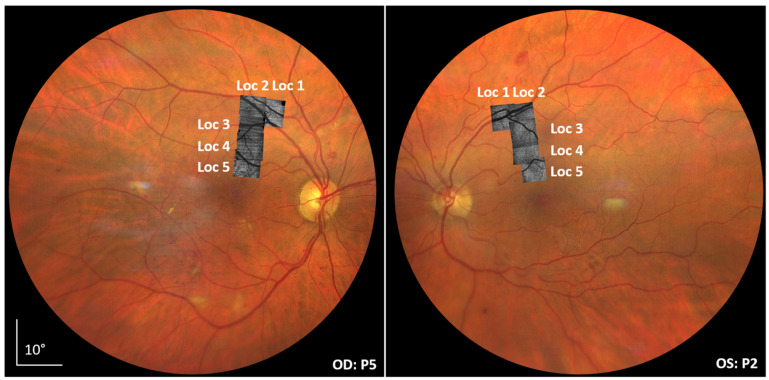
Color fundus images of diabetic retinopathy patients indicating the AO-OCT imaging locations.

For each subject, 2 volumes for a total of 5 focus levels at 5 different retinal locations were recorded. Using this imaging protocol resulted in a relatively low number of volumes, which reduced the overall measurement time and the overall data volume. However, for our analysis, we used only images that are focused on the photoreceptor level. The imaging locations are shown on color fundus photographs in Figure 1. The locations were chosen to cover areas with a different retinal nerve fiber layer (RNFL) thickness and with a varying vessel composition to investigate the potential influence of these structures on the AO-OCT imaging performance. Locations 1–3 show a thick RNFL and a presence of large retinal vessels, while in locations 4–5, the RNFL is thinned and only smaller vessels are present. In total, three young, healthy volunteers (ages 28 y, 29 y, 28 y) and six patients suffering from diabetic retinopathy participated in the study. The volunteers were imaged only once, while each patient was imaged 7–8 times over a period of 12 months. One imaging session, including subject alignment, lasted 20–30 min. All measurements were performed by the same two operators (one for instrument operation, one for subject alignment) and under the same low-light conditions in the lab. Subject alignment relied on an external pupil camera with a standardized alignment procedure. Subjects were allowed to rest between measurements if needed and were asked to blink shortly before each measurement. The characteristics of the patient population are summarized in Table 1. The inclusion criteria of patients for this study were acceptable media opacities and fixation capabilities. All patients showed negligible distortion of the outer retinal layers.

All measurements were performed in accordance with the tenets of the Declaration of Helsinki and after approval by the local ethics committee (EK Nr: 1244/2014). Informed consent was obtained from each participant after explaining the nature and risks of the measurements. Prior to the measurements, 0.5% tropicamide (Mydriaticum Agepha, Vienna, Austria) and 2.5% phenylephrine hydrochloride were administered to enlarge the pupil size. This procedure was not necessary for one healthy subject that already had a pupil size larger than 6.5 mm under the dim light conditions in the lab during data acquisition.

### 2.1. Assessment of AO-OCT Image Quality

**Figure 2:**
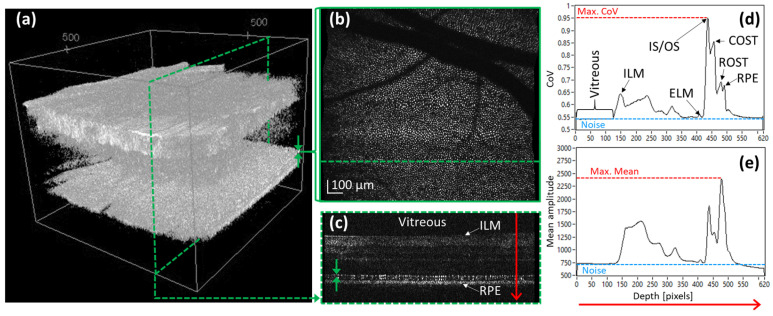
Image data of a healthy volunteer and processing steps for estimating the image quality in AO-OCT. (**a**) Recorded and motion-corrected volume data (green dashed line indicates the position of the B-scan shown in (**c**), arrows indicate the integration depth to generate the en-face scan shown in (**b**)). (**b**) Extracted en-face image of the photoreceptors where *CoV* and mean are calculated. (**c**) Extracted B-scan (green arrows indicate the depth integration range for the en-face scan, the red arrow indicates the direction of the *CoV* evaluation). (**d**) Depth profile of *CoV* values. (**e**) Depth profile of mean values. ILM: inner limiting membrane, ELM: external limiting membrane, IS/OS: junction between inner and outer segments of photoreceptors, COST: cone outer segment tips, ROST: rod outer segment tips, RPE: retinal pigment epithelium.

The acquired AO-OCT volume data were processed according to standard OCT processing [23,24], and B-scans were aligned to each other in the x and z directions, respectively, before further processing (cf. Figure 2). The accuracy of the axial motion correction lies below the axial resolution of the system and is estimated to be ~1–2 µm. The axial position of the central B-scan was stored and used as reference to the axial position of the volume scan. To quantify the achieved AO-OCT image quality, we used two different image quality metrics. First, we calculated the coefficient of variance (*CoV*) for each en-face plane of the volume that is defined as

(1) $$CoV=\frac{\sigma }{\langle A\rangle },$$

where σ represents the standard deviation and $\langle A\rangle$ the mean of the OCT amplitude value within an en-face image. The result is a depth profile of *CoV* values (cf. Figure 2d). This metric considers the contrast of cellular retinal structures, specifically of the photoreceptor mosaic, and has been used to estimate the image quality in AO-SLO [22]. We then used the maximum of this depth profile and the noise floor (that was measured in the vitreous to be 0.55) for further evaluation. The noise floor was the same for all subject and patient measurements. Second, we calculated the mean of the OCT amplitude within each en-face imaging plane in a similar way. The result is a depth profile of the mean amplitude $\langle A\rangle$ showing the highest peak around the photoreceptor layers (cf. Figure 2e).

Interestingly, the axial location of the maximum differs slightly between the *CoV* and mean depth profiles. We found that the *CoV* depth profile represents a better measure of the sharpness of the individual layers of the retina and, as can be seen in Figure 2d), it allows for a clearer separation between the photoreceptor layers. Furthermore, in our spectral domain system, the sensitivity depends on the axial position of the structure with respect to the zero delay (between reference and sample arm, respectively). Measurements in the model eye showed (see Supplementary Figure S1) that the influence of the sensitivity is much more pronounced for the mean value than for the *CoV* value. Thus, we decided to use only the maximum *CoV* values for further analysis.

**Figure 3:**
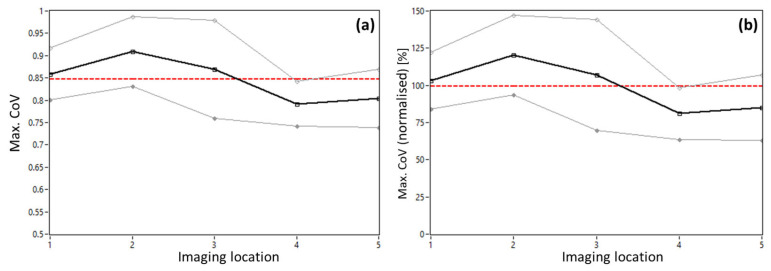
AO-OCT image quality in healthy subjects. (**a**) Maximum *CoV* value along the imaging depth with dependence on the imaging location (black line: average over 3 healthy subjects, grey lines: standard deviation, red line: average over all imaging locations). (**b**) Maximum *CoV* value normalized to the average over all healthy volunteers and locations, in percent.

For a better interpretation of the derived *CoV* values, we used the image data retrieved from the 3 healthy volunteers as a reference. Figure 3 shows the averaged *CoV* values (±standard deviation) of the healthy volunteers for the 5 different imaging locations. The average *CoV* value over all healthy subjects and all imaging locations (cf. red line in Figure 3a) was used to normalize the data after subtraction of the noise floor value.

(2) $$Max.CoV\;\left(normalised\right)=\frac{{Max.CoV}_{Patient}-0.55}{{Max.CoV}_{Average\;Healthy}-0.55}\times 100,$$

The result is a graph that shows the AO-OCT image quality in reference to the achieved mean image quality in healthy volunteers in percent (cf. Figure 3b).

### 2.2. Evaluation of Pyramid Wavefront Sensor Data

**Figure 4:**
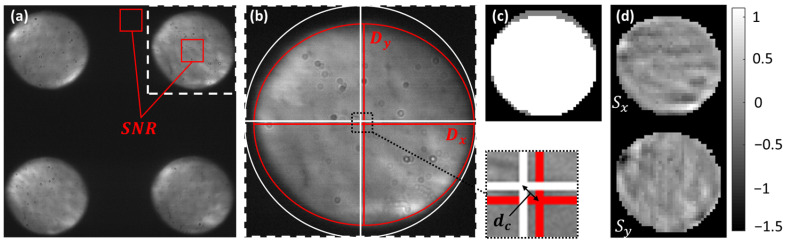
Pyramid wavefront sensor data retrieved in a healthy volunteer after AO loop convergence. (**a**) Full pyramid wavefront sensor image indicating the region of interest (white square) that was used for further evaluation. Regions that were used to calculate the signal-to-noise ratio of the pyramid wavefront sensor images are indicated by red squares. (**b**) Region of interest, indicating the central pupil position and pupil size of the system in white and the measured central pupil position and size in red. d_c_ denotes the deviation of the pupil from the central position. D_x_ and D_y_ denote the diameter of the pupil in the x and y directions, respectively. (**c**) Pixels above a certain intensity threshold (after pixel binning) that are used to calculate the wavefront slopes. White: detected pixels of in vivo measurement, grey: detected pixels in the model eye corresponding to the system pupil size. (**d**) Retrieved wavefront slopes in the x and y directions, respectively.

The pyramid wavefront sensor yields 4 images of the pupil plane of the eye (cf. Figure 4a). From the intensity distribution between the images, the wavefront slopes can be derived (cf. Figure 4d) [14,25,26]. The pyramid wavefront sensor images that we used for our analysis were stored after AO-loop convergence and up to ~20 s after the corresponding AO-OCT volume recording.

We derived various metrics for our analysis in order to compare these to the AO-OCT image quality. These include the signal-to-noise ratio of the pyramid sensor image (SNR_p_), which we defined as the ratio between the two areas (indicated in red in Figure 4a). The diameter D of the pupil that was automatically detected in the region of interest was calculated as the average between the diameters in the x and y directions, respectively (cf. Figure 4b). The displacement d_c_ of the pupil is given by the difference of the measured pupil position to the central pupil position of the system. Finally, we calculated the AO correction quality AO_q_. The basis for the calculation of AO_q_ is the root mean square (RMS) value of the residual slopes (cf. Figure 4d) of the pyramid wavefront sensor in the x and y directions, respectively. The calculation of these slopes is described in previous work [12]. As the RMS strongly depends on the pupil size, we normalize the RMS value by the pupil area (number of white pixels $N_{P}$ in Figure 4c) and calculate the inverse function as follows:

(3) $${AO}_{q}=\frac{N_{P}}{RMS}.$$

This ensures that AO_q_ is a value that increases with better AO correction (lower RMS) and larger pupil areas.

## 3. Results

### 3.1. AO-OCT Image Quality in Patients

**Figure 5:**
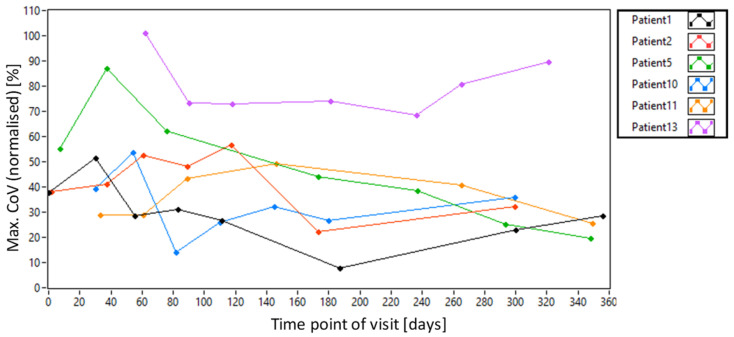
Dependence of AO-OCT image quality on the imaging day for all patients, estimated by normalized *Max.CoV* values.

**Figure 6:**
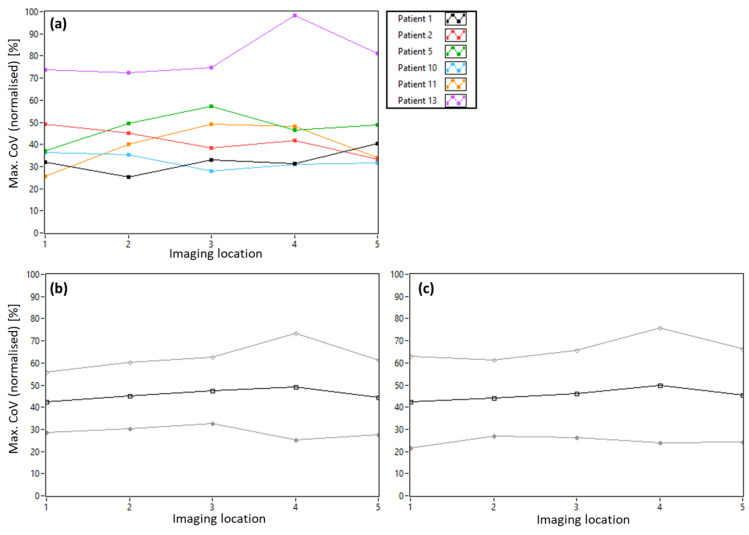
Dependence of AO-OCT image quality on the imaging location estimated by normalized *Max.CoV* values. (**a**) *Max.CoV* values for each patient averaged over all visits. (**b**) Mean (black line) and standard deviation (grey lines) of *Max.CoV* values for all patients for volume 1 and (**c**) volume 2.

The normalized *Max.CoV* values for our patient population averaged over all imaging locations are shown in Figure 5. Representative images of the extreme cases (highest and lowest *CoV* values) are provided in Figure S2 of the Supplementary Materials. The large variance in AO-OCT image quality between patients and visits is evident. In addition, a much lower quality (~40% compared to healthy subjects) can be observed than what can be achieved, on average, in healthy subjects. Figure 6a) shows the data averaged over all visits but split into the five imaging locations (the data in Figure 3b are used as a reference). Although there are variations between the locations for each patient, the average over all patients shows a rather stable AO-OCT image quality for all imaging locations and insignificant differences between the two recorded volumes (cf. Figure 6b,c). This indicates that the imaging location has a negligible influence on the AO-OCT image quality at the photoreceptor level despite the structural differences of the inner retinal layers.

### 3.2. Pyramid Wavefront Sensor Data in Patients

**Figure 7:**
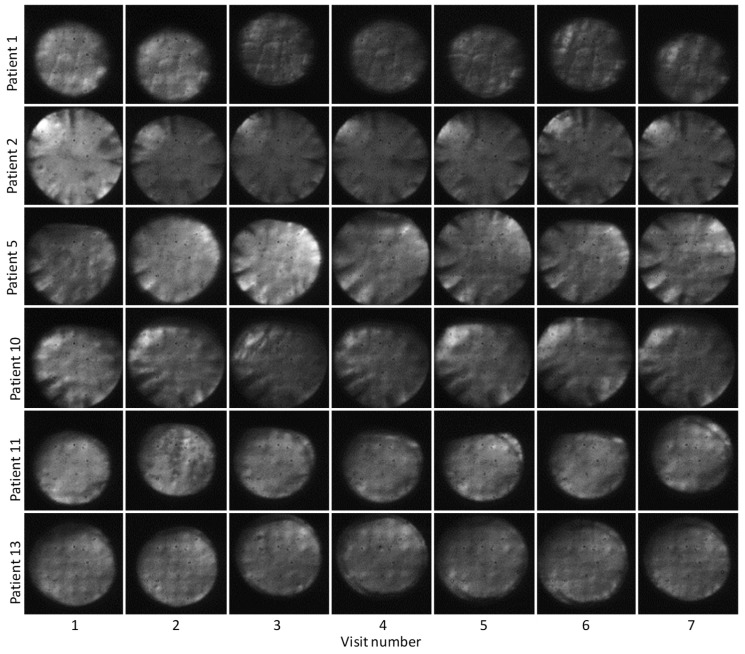
Representative pupil images recorded at location 2 for all patients and all visits.

Figure 7 gives an overview of the pupil images that have been recorded at location 2 for all patients and all visits. Although the pupil size varies between patients, the size from visit to visit remains rather constant for each patient. Patients with natural lenses (Patient Nr. 2,5,10) show dark wedges in the periphery of the pupil images that are not present in the healthy eye (cf. Figure 4) nor in the eyes with an artificial lens. These wedges are visible at all visits and are likely caused by early-stage cortical cataract in these patients. For patients 11 and 13, the outer boundary of the artificial lens can be seen as a dark ring. The appearance of a very regular pattern in the pupil images of patients 11 and 13 is noteworthy. This pattern is caused by the elements of the DM and typically indicates a very good AO-correction performance because the residual wavefront error is then dominated by the discrete elements of the DM.

**Figure 8:**
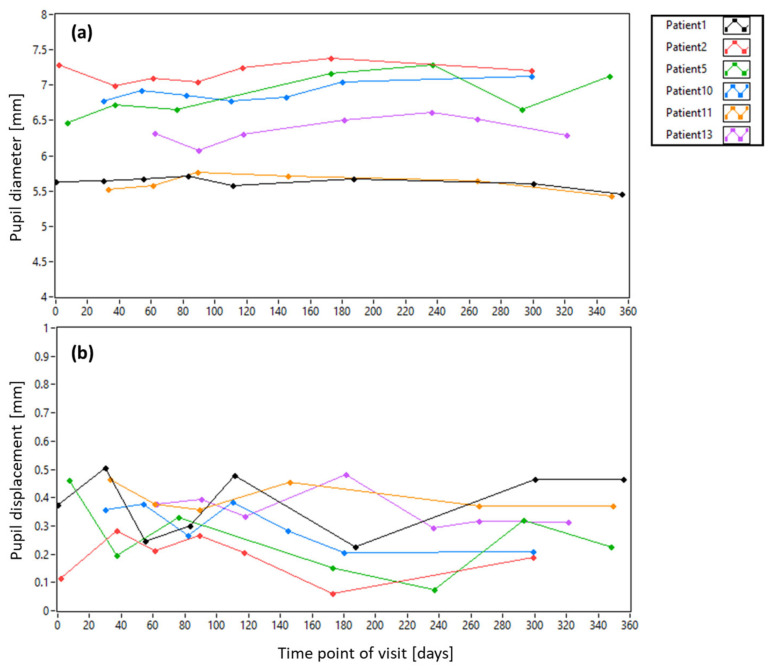
Dependence of pupil diameter (**a**) and pupil displacement (**b**) on the imaging day for all patients.

**Figure 9:**
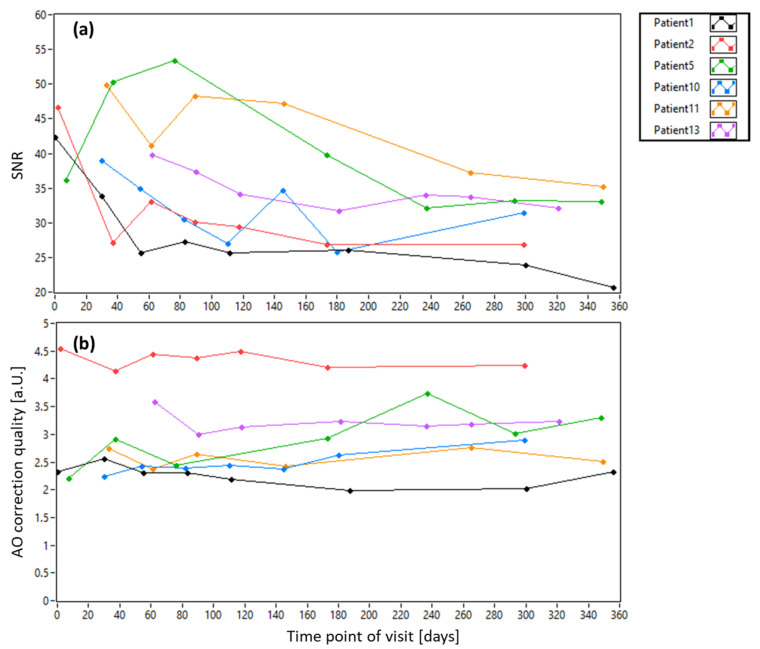
Dependence of SNR of the pupil images (**a**) and AO-image quality (**b**) on the imaging day for all patients.

Figure 8 shows the pupil diameter and the pupil displacement of all patients with dependence on the imaging day (the dependence on imaging location is shown in the Supplementary Materials Figure S3). While the pupil diameter stays very similar from visit to visit, the lateral deviation of the pupil center from the center of the system shows a large variability (cf. Figure 8b). On average, the alignment of the subjects yielded a deviation from the central pupil position of <200 µm and <350 µm for healthy subjects and patients, respectively (cf. Supplementary Materials Figure S4). Figure 9 shows the SNR of the pupil images and the AO correction quality with dependence on the imaging day. (The dependences on imaging location are displayed in Supplementary Figure S5 and Figure S6, respectively). A slight trend to lower SNR at later visits can be observed in the data, possibly introduced by a progression of cataract. The AO correction quality differs from patient to patient and shows some variability from visit to visit. However, no general trend can be observed in these data.

**Figure 10:**
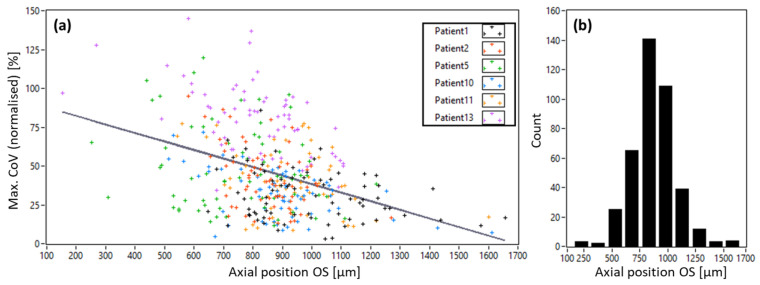
(**a**) Correlation plot between axial position of the outer segments of cones (OS) with respect to the zero delay of the system and the normalized *CoV* values. A Pearson coefficient of −0.41 (*p* < 0.01) was found, and the black line indicates a linear fit of the data. (**b**) Distribution of axial positions of the OS for all volume recordings.

To detect potential relations between our AO-OCT image quality metric and the axial position in the image, we show a scatter plot of all volume recordings in Figure 10. The axial position of a volume was derived from the position of the outer segments of cone photoreceptors (dark band) in the central B-scan image of the volume acquisition with respect to the zero delay line. Although the *CoV* values are less sensitive to the sensitivity roll-off of the system (see Supplementary Figure S1), a clear dependence of the *CoV* values on the axial position can be observed. The mean axial position of all measurements was found to be (806 ± 194) µm and indicates a rather stable axial position for all measurements.

**Figure 11:**
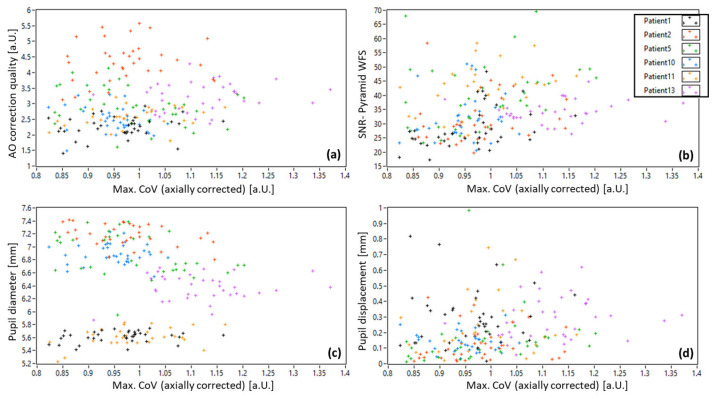
Correlation plots of patient data between *Max.CoV* values and (**a**) AO-correction quality, (**b**) SNR of the pyramid wavefront sensor, (**c**) pupil diameter and (**d**) pupil displacement from the central position. The Pearson coefficient was measured to be 0.11 (*p* = 0.12), 0.24 (*p* < 0.01), −0.06 (*p* = 0.43) and 0.2 (*p* < 0.01), respectively.

For the further analysis of potential correlations between AO-OCT image quality and wavefront sensor data, we corrected all *CoV* values according to the corresponding axial position using the linear regression line in Figure 10. Figure 11 shows plots between the axially corrected AO-OCT image quality and the calculated AO correction quality, the SNR of the pyramid sensor image, the pupil diameter and the displacement of the pupil center from the central position for all patients. For all these data, we calculated the Pearson coefficient to investigate potential correlations. Surprisingly, the pupil diameter does not show a linear correlation with AO image quality. A very weak correlation was found between AO-image quality and AO-correction quality, as well as between AO-image quality and pupil displacement. A weak correlation was found between AO-image quality and SNR of the pupil images, as well as AO-image quality and pupil displacement.

## 4. Discussion

Our longitudinal study of the AO-OCT image quality in patients with diabetic retinopathy reveals largely varying imaging results between patients and between visits of the same patient (cf. Figure 5). Part of this variability results from the different axial positions of the volume recordings (cf. Figure 10a). This dependency is caused by the sensitivity roll-off [27] of our spectral domain instrument and needs to be considered when evaluating potential other influences on the image quality, such as the AO correction performance. The influence on axial position can be minimized, for example, by the use of a swept source OCT system. These systems typically show a lower sensitivity roll-off, enabling large imaging depths [28]. However, these instruments are operated in the 1060 nm wavelength band and, therefore, provide a lower transverse resolution than instruments at 840 nm. Another possibility is the implementation of axial retinal tracking [29]. Ideally, such tracking is fast enough to stabilize the axial position of each B-scan. However, this requires a significant amount of additional equipment [6]. Lower speeds for axial eye motion correction might be sufficient to reduce this influence and can be realized by taking the axial position of each B-scan as a parameter to adjust the reference arm mirror position accordingly. Although our results indicate some dependence of the image quality on the axial position, there are other factors (such as the SNR of the pyramid wavefront sensor) that need to be considered as well.

Using the pyramid wavefront sensor, we retrieved various parameters, such as AO-correction quality, SNR of the pupil images, pupil diameter and pupil displacement from the central position. These parameters were then tested for potential correlations with the axially corrected AO-image quality and only a weak (but significant) correlation with the SNR of the pupil images and with the lateral displacement of the pupil was found (cf. Figure 11).

The weak correlation of the SNR is potentially caused by the transparency of the ocular media of a patient. Lower transparency (introduced, for example, by early cataract) will naturally result in lower SNR of the pupil images as well as of the AO-OCT images. Similarly, this weak correlation may be induced by varying scattering properties of the retina. However, the results indicate that other factors contribute more to the AO-OCT image quality in our patient population than the transparency of the ocular media or the scattering properties of the retina.

The weak correlation of the lateral pupil displacement with the AO-OCT image quality (cf. Figure 11d) is puzzling because it indicates that a larger displacement results in better quality. Obviously, there must be an upper limit when the displacement results in pupil clipping, but this limit seems not to be reached in our data. Otherwise, for smaller pupils, the exact lateral positioning of the pupil in respect to the system does not seem to degrade the AO-OCT image quality.

Interestingly, our data show that the pupil diameter and the AO-correction quality have a low (and nonsignificant) impact on the final AO-OCT image quality. This seems to be in contradiction to the assumption that larger pupil size and lower RMS values result in better image quality. As an increase in the pupil diameter from 5.5 mm to 7.5 mm roughly translates into a doubling of the pupil area size, the amount of light returning from the retina should be doubled as well, thus significantly increasing the AO-OCT image quality.

However, the demands on the AO-correction increase with pupil diameter. In our case, because we are imaging on a 4° × 4° field of view, we need to consider variations of aberrations across the field of view or, in other words, the limited size of the isoplanatic patch as well [30]. While varying aberrations can partly be compensated along the slow scanning direction using a fast AO-correction loop [14], our wavefront sensing (and thus AO-correction) is rather insensitive to varying aberrations across the fast scanning direction. This results in an AO-correction that does not account for the varying aberrations across the field of view. The presence of residual wavefront aberrations that are not detectable by our sensor degrades the imaging performance on this larger field of view, thus counteracting the benefit of increased light power returning from the retina.

According to our data, there seems to be a “sweet spot” of around 6.5 mm pupil diameter (cf. patient 13) that yields the best image quality across the field of view. However, as only one patient falls into this pupil diameter, this observation needs to be considered with care. To achieve better AO-OCT image quality results in larger pupils, the varying aberrations across the field of view need to be compensated, for example, by implementing a second deformable mirror [31].

An interesting aspect is that in patients, the precision of manually aligning the pupil center with our standard head rest is better than 360 µm (cf. Figure S3). As we do not observe a strong correlation of the deviation from the pupil center with the AO-OCT image quality, this precision seems sufficient. Thus, the demands for automatic pupil tracking, as demonstrated earlier [32] in AO-OCT systems, are not very high.

The observed varying image quality between imaging locations in individual patients (cf. Figure 6) might be caused, for example, by structural changes in the inner retina (hard exudates, etc.) that cause shadowing on the photoreceptor layer. However, the average of the data over all patients does not show a dependence on imaging location. This indicates that our wavefront sensing is marginally influenced by the varying structure of the anterior retinal layers.

One limitation of our study is the small patient population and the small group of healthy subjects. However, our data represent a first attempt to find a link between wavefront sensor-derived data and AO-OCT imaging quality. As no high correlation was found, it emphasizes the need for additional metrics in order to understand the relationship between those two metrics. One parameter could be the final shape of the correcting device by storing the DM shape. Another limitation of our study is that the wavefront sensor data were not simultaneously recorded with the AO-OCT data. Although the time separation is only a few seconds, some uncertainties remain that may influence the measured correlation between the parameters. Finally, we did not include an analysis of the axial motion within a volume recording. In cases of large axial motion, this could potentially lead to a deterioration of the measured image quality in both directions (better or poorer). Although in our analysis such influence will be averaged out (cf., for example, Figure 6b,c, which do not show a difference between the recordings of volumes 1 and 2), it might be worthwhile to look into this influence in more detail in future studies.

The widespread use of our introduced image quality metrics for AO-OCT would potentially enable better comparison between the imaging performances of various instruments. On the long road towards the establishment of AO-OCT in clinical routine, such comparison represents an essential step. Another aspect is that these image quality metrics can be used for automatically provided feedback to the instrument operator on the achieved image quality. This aids the operator in the decision to retake a measurement or not. However, this requires a fast graphic processing unit (GPU)-based evaluation of the data that we have not currently implemented.

## 5. Conclusions

We introduced imaging metrics for assessing the AO-OCT image quality and compared these with parameters retrieved by our pyramid wavefront sensor. The longitudinal analysis in patients with diabetic retinopathy revealed high variability in image quality between subjects and within visits of the same subject. On average, the choice of the specific imaging location does not influence the image quality. Even though in sub-millimeter range, changes in the axial position have a larger influence on the image quality than lateral pupil displacements. Our study emphasizes the importance of the simultaneous monitoring of image quality and wavefront sensor data to further improve AO-OCT imaging performance in patients.

## Figures and Tables

**Table 1:** Characteristics of the patient population that was included in this study. The ocular condition was assessed at baseline.

Patient	Eye	Age	Sex	DM Type	DR Stage	BCVA	Ocular Condition	Lens Status
**1**	OS	79	F	II	Moderate npDR	20/20	DME, PCO inc.	Artificial
**2**	OS	57	M	II	Moderate npDR	20/25	DME, Cat.	Natural
**5**	OD	67	M	II	Severe npDR	20/32	DME, Cat., Dry eye	Natural
**10**	OS	66	M	II	Moderate npDR	20/32	DME, Cat.	Natural
**11**	OD	81	M	II	Mild npDR	20/20	DME	Artificial
**13**	OD	67	M	II	Severe npDR	20/32	DME	Artificial

(np)DR: (nonproliferative) diabetic retinopathy. BCVA: Best corrected visual acuity. DME: Diabetic macular edema. PCO: Posterior capsule opacification. inc: incipient. Cat: Cataract.

## Data Availability

Data underlying the results presented in this paper are not publicly available at this time but may be obtained from the authors upon reasonable request.

## Supplementary Materials

The following supporting information can be downloaded at: https://www.mdpi.com/article/10.3390/diagnostics15040429/s1, Figure S1: Measurements taken from the scattering surface of the model eye to show the dependence of the maximum *CoV* value (a) and mean value (b) on the axial position; Figure S2: Representative images of diabetic patients recorded at imaging location 4 illustrating the range of image quality (best to poor); Figure S3: Pupil diameter in dependence of the imaging location for our study population; Figure S4: The displacement d_c_ in dependence of the imaging location for our study population; Figure S5: The signal to noise ratio (SNR) of the pupil images in dependence on the imaging location; Figure S6: AO-correction quality in dependence on the imaging location; Table S1: Additional characteristics of the patient population.
