# Supplementary material for: Image Quality in Adaptive Optics Optical Coherence Tomography of Diabetic Patients
Source: Diagnostics (Basel). 2025 Feb 10;15(4):429. doi: 10.3390/diagnostics15040429 (PMC11854792; doi:10.3390/diagnostics15040429)
Supplement: Supplementary file 1 [file diagnostics-15-00429-s001.zip › diagnostics-3377422-supplementary.pdf]

Supplementary Materials

Image Quality in Adaptive Optics Optical Coherence Tomography Imaging of Diabetic Patients

Elisabeth Brunner[1], Laura Kunze[2], Wolfgang Drexler[1], Andreas Pollreisz[2] and Michael Pircher[1,*]

Figure S1 shows the dependence of AO-OCT image quality metrics on the axial postion, as measured in the model eye. Figure S2, illustrates the image quality in diabetic patients for two extreme cases (best and lowes CoV values. Figure S3, shows the pupil diameter of all subjects in dependence on the imaging location. The patient data is averaged over all visits. On average a smaller pupil diameter can be observed in patients in comparison with the healthy control group. The deviation of the pupil center from the center of the system is plotted in Fig. S4. On average, the alignment of the subjects yielded a deviation from the central position of 200µm and 350µm for healthy subjects and patients, respectively. Figure S5 shows the SNR in dependence on the imaging location. Here a clear trend can be observed for both healthy and patient data. The SNR is lower for imaging locations with lower eccentricity from the fovea. This observation can possibly be explained by the differing retinal structure at these locations. In the presence of a thicker retinal nerve fiber layer, more light will be detected by the pyramid wavefront sensor which will contribute to the measured SNR. Figure S6 shows the AO-correction quality in dependence of the imaging location. Table S1 provides additional data on the subjects eye length and refractive power.

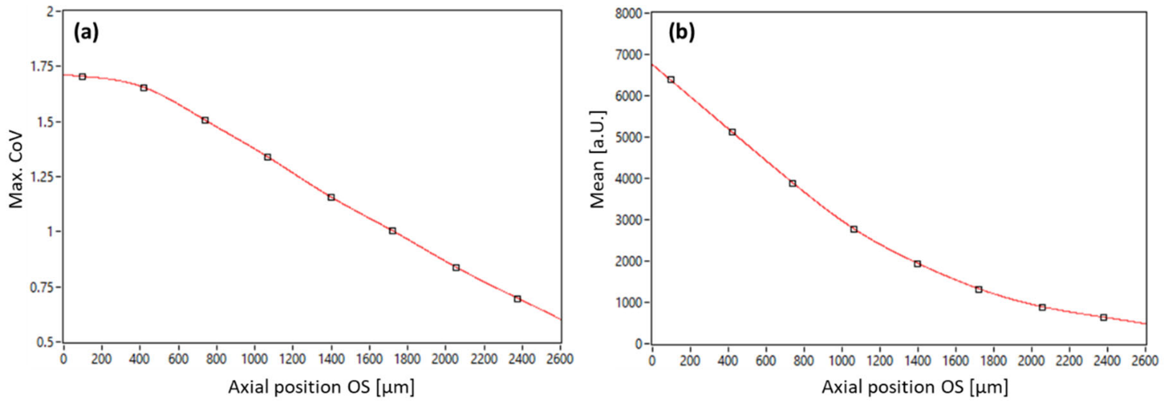

Figure S1. Measurements taken from the scattering surface of the model eye to show the dependence of the maximum CoV value (**a**) and mean value (**b**) on the axial position.

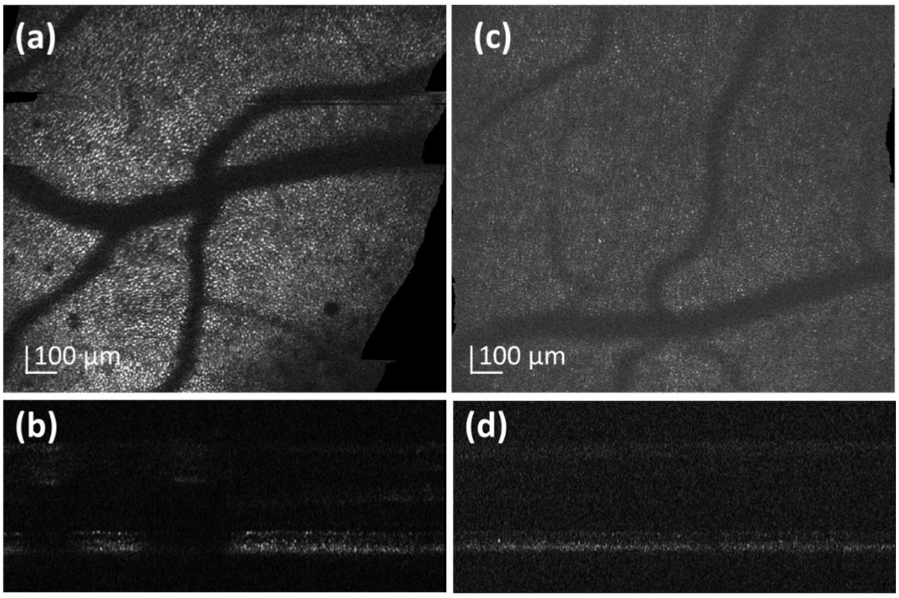

Figure S2. Representative images of diabetic patients recorded at imaging location 4 illustrating the range of image quality (best to poor). (**a**) en-face image, and (**b**) central B-scan image of patient 13 recorded at visit 1 (highest CoV value). (**c**) en-face image, and (**d**) central B-scan of patient 1, visit 6 (lowest CoV value).

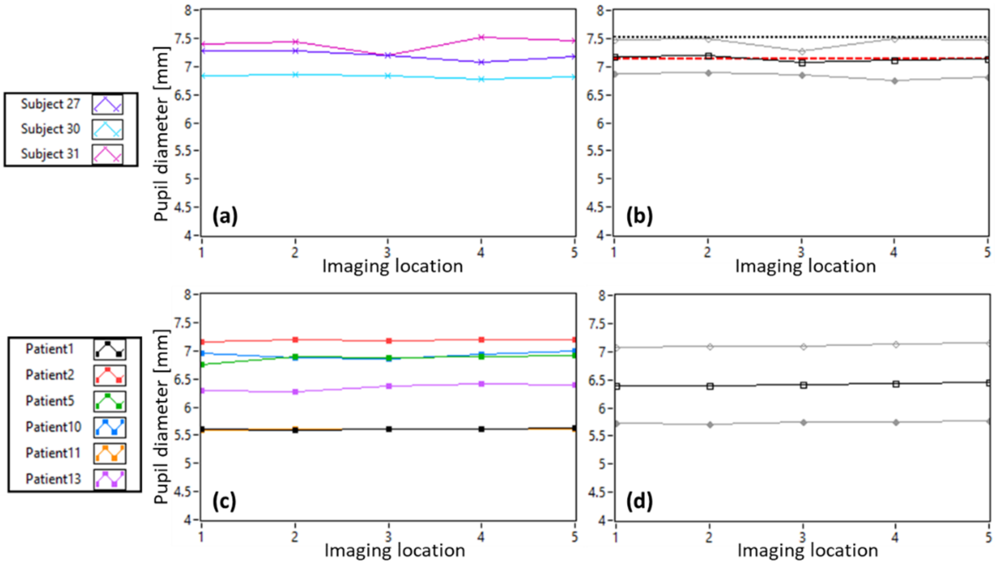

Figure S3. Pupil diameter in dependence of the imaging location for our study population. (**a**) Healthy subjects, each color represents a different subject. (**b**) Averaged data over all healthy subjects. The black line indicates the mean value, the grey lines indicate the mean ± standard deviation. The dashed black line indicates the pupil size of the system, the dashed red line indicates the mean value over all imaging locations. (**c**) Patient data averaged over all visits (each color represents the data of a patient). (**d**) Mean pupil diameter of all patients (black line) and mean ± standard deviation (grey lines).

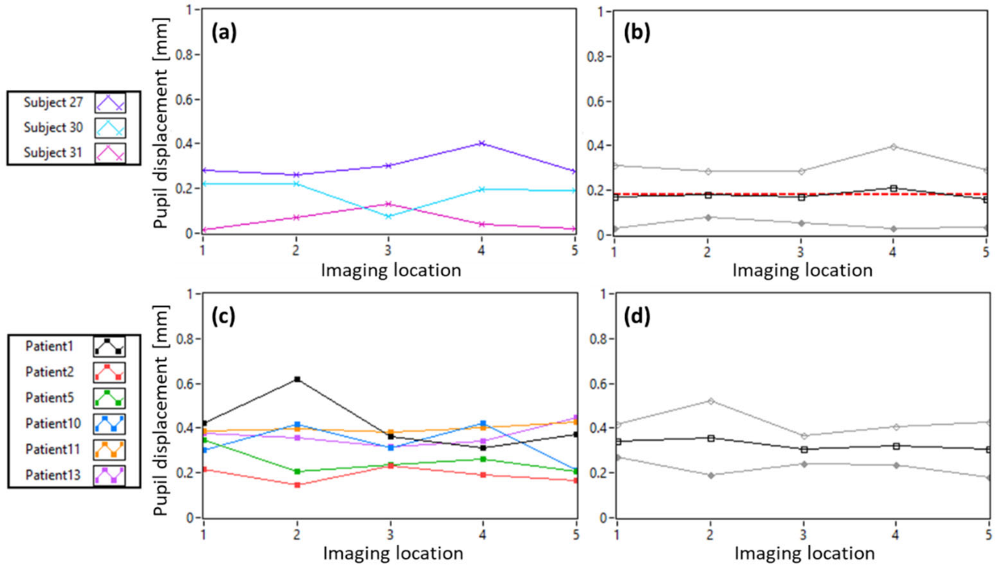

Figure S4. The displacement d_c in dependence of the imaging location for our study population. (**a**) Healthy subjects, each color represents a different subject. (**b**) Averaged data over all healthy subjects. The black line indicates the mean value, the grey lines indicate the mean ± standard deviation. The dashed red line indicates the mean value over al imaging locations (**c**) Patient data averaged over all visits (each color represents the data of a patient). (**d**) Mean pupil displacement of all patients (black line) and mean ± standard deviation (grey lines).

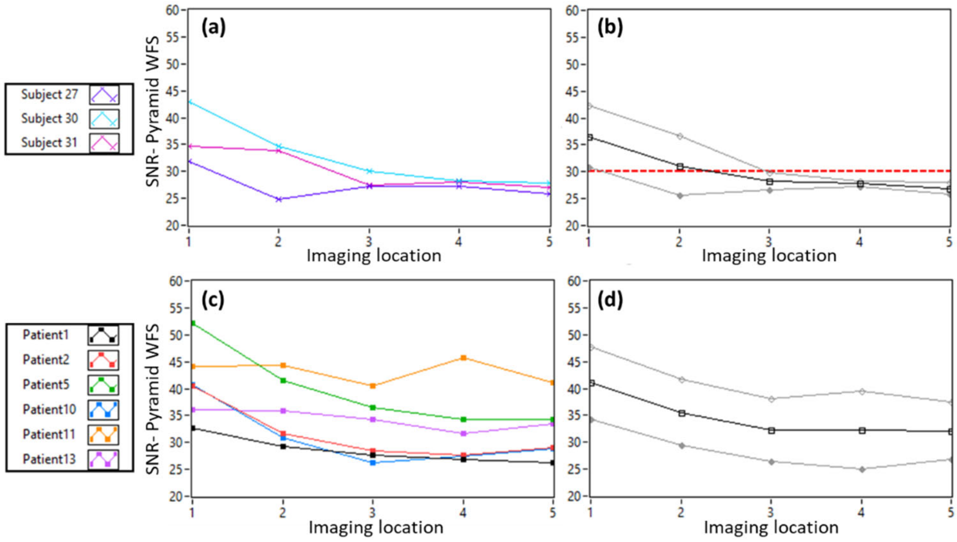

Figure S5. The signal to noise ratio (SNR) of the pupil images in dependence on the imaging location. (**a**) Healthy subjects, each color represents a different subject. (**b**) Averaged data over all healthy subjects. The black line indicates the mean value, the grey lines indicate the mean ± standard deviation. The dashed red line indicates the mean value over all imaging locations. (**c**) Patient data averaged over all visits (each color represents the data of a patient), (**d**) Mean SNR of all patients (black line) and mean ± standard deviation (grey lines).

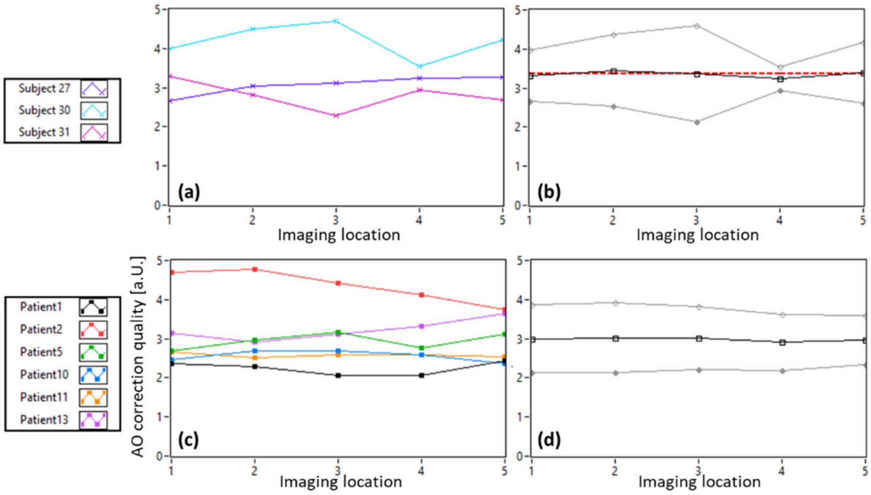

Figure S6. AO-correction quality in dependence on the imaging location. (**a**) Healthy subjects, each color represents a different subject. (**b**) Averaged data over all healthy subjects. The black line indicates the mean value, the grey lines indicate the mean ± standard deviation. The dashed red line indicates the mean value over all imaging locations. (**c**) Patient data averaged over all visits (each color represents the data of a patient), (**d**) Mean AO correction quality of all patients (black line) and mean ± standard deviation (grey lines).

Table S1. Additional characteristics of the patient population

Patient (eye)	Eye length / mm	Anterior chamber depth / mm	Refractive error (spherical, astigmatism, axis)
P1 (OS):	22.31	2.96	-0.5, +2.0, 172
P2 (OS):	25.14	3.53	-0.75, +1.75, 49
P5 (OD):	23.84	3.1	+1.0, +0.5, 32
P10 (OS):	23.25	2.97	+0.25, +0.25, 91
P11 (OD):	23.12	3.47	-1.5, +1.5, 22
P13 (OD):	24.21	5	-0.75, +0.75, 5
